# MicroRNA-761 suppresses remodeling of nasal mucosa and epithelial–mesenchymal transition in mice with chronic rhinosinusitis through LCN2

**DOI:** 10.1186/s13287-020-01598-7

**Published:** 2020-04-09

**Authors:** Jinzhang Cheng, Junjun Chen, Yin Zhao, Jingpu Yang, Kai Xue, Zonggui Wang

**Affiliations:** 1 Department of Otolaryngology Head and Neck Surgery, the Second Hospital of Jilin University, No. 218, Ziqiang Street, Nanguan District, Changchun, 130041 Jilin Province People’s Republic of China; 2 Department of Pharmacy, the Second Hospital of Jilin University, Changchun, 130041 People’s Republic of China

**Keywords:** Chronic rhinosinusitis, MicroRNA-761, Lipocalin 2, Nasal mucosa, Epithelial–mesenchymal transition

## Abstract

**Background:**

Chronic rhinosinusitis (CRS) is characterized by persistent symptomatic inflammation of the nasal passage and sinus mucosa. Various microRNAs (miRs) have been implicated in CRS. Hence, the current study was conducted to explore the effect of microRNA-761 (miR-761) on remodeling of nasal mucosa and epithelial–mesenchymal transition (EMT).

**Methods:**

Bioinformatics analysis was initially performed to predict the differentially expressed genes (DEGs) associated with CRS. Gene targeting relationship between miR-761 and lipocalin 2 (LCN2) was analyzed by bioinformatics analysis and verified using dual-luciferase reporter gene assay. Histopathological analyses of the nasal mucosa tissues were conducted via hematoxylin–eosin (HE) and alcian blue (AB)-periodic acid Schiff (PAS) staining. ELISA was employed to determine the IL-8 and MMP-9 levels. To define downstream pathway of miR-761, levels of proteins related to LCN2/Twist1 signaling pathway were assessed. Additionally, the effects of miR-761 on EMT, proliferation, and apoptosis were determined.

**Results:**

LCN2 was highly expressed in CRS. LCN2 was a target of miR-761. miR-761 overexpression or LCN2 silencing decreased IL-8 and MMP-9 levels and morphological changes in nasal epithelial tissue from CRS mice. Overexpressed miR-761 or silenced LCN2 decreased the expression of LCN2 and Twist1, indicating LCN2/Twist1 signaling pathway was inactivated. Moreover, miR-761 overexpression or LCN2 silencing reduced the expression of N-cadherin and vimentin, while increased that of E-cadherin, suggesting inhibition of EMT. Furthermore, miR-761 overexpression or LCN2 silencing promoted cell proliferation and inhibited cell apoptosis in CRS.

**Conclusion:**

Taken together, miR-761 suppressed the remodeling of nasal mucosa through inhibition of LCN2 and the LCN2/Twist1 signaling pathway.

## Background

Chronic rhinosinusitis (CRS) is a chronic inflammatory disorder characterized by chronic nasal obstruction, hyposmia, and facial pain [1]. The cause of CRS has been widely speculated to be triggered by environmental factors, perennial allergic rhinitis, and gastroesophageal reflux [2]. Patients suffering from CRS commonly experience reduced sleep, decreased cognitive function, anxiety, and depression [3]. Remodeling of nasal mucosa is involved in the progression of nasal polyp and is caused by persistent inflammation [4]. Moreover, the epithelial–mesenchymal transition (EMT) is a feature of CRS with nasal polyps [5]. Typically, CRS strategies are predominately aimed at alleviating mucosal inflammation, preventing infection, and clearing mucus within the sinuses [6]. There are various treatment options for CRS, including the use of oral antihistamines as well as antibiotics [7]. However, patients suffering from CRS often complain of recurrence of symptoms even following pharmacological and surgical treatment [8]. Therefore, more effective treatments with minimal side effects capable of reducing the rate of CRS recurrence are of urgent need.

Lipocalins (LCN) represent a protein family of approximately 20 members. LCN has diverse biological functions, including immune reaction regulation, cell growth and metabolism, iron transport, and prostaglandin synthesis [9, 10]. As a member of the LCN family, lipocalin 2 (LCN2) has been shown to play a role in the genetic regulation of signaling pathways related to cytokine-mediated cell growth and inflammatory responses [11]. LCN2 is downregulated by microRNA-138 (miR-138), suppressing apoptosis in cardiomyocyte triggered by hypoxia [12]. MicroRNAs (miRs) have been widely implicated in a variety of physiological and pathological activities including proliferation, differentiation, inflammatory reaction, and tumorigenesis [13, 14]. Various miRs, including miR-125b and miR-26a, are involved in the inflammatory and immunological reactions observed in CRS [15]. However, studies concerning the involvement of the less characterized miR-761 from a CRS perspective remain unknown. Furthermore, Twist1 is a helix–loop–helix transcription factor responsible for enhancing EMT, invasiveness, and metastasis in many tumor cells [16, 17]. The relationship between LCN2 and Twist1 has been previously reported that the LCN2/Twist1 signaling pathway negatively regulates EMT in hepatocellular carcinoma [18]. Based on these previous findings, we aim to explore how the remodeling of nasal mucosa and EMT in a mouse model of CRS is affected by miR-761, hoping to discover a potential therapeutic biomarker.

## Methods

### Ethical statements

The study was conducted in strict accordance with the recommendations of the Guide for the Care and Use of Laboratory Animals from the National Institutes of Health. All protocols were approved by the Institutional Animal Care and Use Committee of the Second Hospital of Jilin University.

### Microarray analysis

CRS gene expression chips were retrieved from the Gene Expression Omnibus (GEO) database (https://www.ncbi.nlm.nih.gov/geo/) using the keyword “chronic rhinosinusitis.” The outcome exploration process was fine-tuned by narrowing the study type and organism to “expression profiling by array” and “*Homo sapiens*.” GSE10406 chip, including expression data in CRS tissues from 8 normal controls and 8 CRS patients, was selected for the analysis of differentially expressed genes (DEGs). The Limma package in the R Language Programming was employed for microarray gene expression pretreatment and DEG screening with |LogFoldChange| > 2, with the screening threshold expressed as adj.P.Val (*p* value after correction). The heat maps of the DEGs were subsequently constructed. DigSee (http://210.107.182.61/geneSearch/) was used to identify MEDLINE abstracts with the keyword “chronic rhinosinusitis” for disease gene information. All differentially expressed genes that were related to CRS were included in the String database (https://string-db.org/) for gene interaction analysis and visualized using Cytoscape 3.6.0 to identify potential key DEGs. The DEGs that were regulated by miRs were predicted by microRNA (http://34.236.212.39/microrna/getGeneForm.do), miRWalk (http://mirwalk.umm.uni-heidelberg.de/), and miRNAMap (http://mirnamap.mbc.nctu.edu.tw/) databases. The obtained results were compared using jvenn (http://jvenn.toulouse.inra.fr/app/example.html).

### CRS animal model establishment

A total of 56 C57BL/6 mice aged 6–8 weeks (weight 18–22 g) were recruited in this study. The mice were randomly divided into the CRS or control groups (*n* = 8). Nasal infection was induced by placing expansive sponge slices into the left nasal cavity (The depth of forceps insertion did not exceed 1.5 mm, and the sponge was slowly inserted into the nasal cavity for 4 mm) containing standard strains of *Staphylococcus aureus* suspension. The bacteria were perfused in the ostiomeatal complex. Control mice did not receive any sponge slice. The CRS mice were further divided into six groups (*n* = 8/group): CRS (CRS mice), negative control (NC) (CRS mice transfected with lentiviral particles in empty vectors), miR-761 mimic (CRS mice transfected with miR-761 overexpressed lentiviral particles), miR-761 inhibitor (CRS mice transfected with miR-761 silencing lentiviral particles), the siRNA against LCN2 (si-LCN2) group (CRS mice transfected with LCN2 silencing lentiviral particles), and the miR-761 inhibitor + si-LCN2 group (CRS mice co-transfected with miR-761 silencing lentiviral particles and LCN2 silencing lentiviral particles). On the seventh day post transfection, each mouse in the miR-761 inhibitor group and the si-LCN2 group was added dropwise with 1 × 10^9^ TU/mL lentiviral particles through the nasal cavity for consecutive 10 days. The lentivirus was purchased from Cyagen Biosciences Inc. (Guangzhou, China). Mice of each group were analyzed and observed individually for 30 min and evaluated by overlap scoring. A score of more than 5 points was considered to be indicative of successful modeling. Additionally, sneezing and nasal rubbing scores were recorded (Table 1). The mice were euthanized 4 weeks after the transfection. Blood and epithelial tissues in the paranasal sinus mucosa were collected. The epithelial tissues were rinsed in physiological saline and fixed with formaldehyde.

**Table 1:** Evaluation and scoring for nasal rubbing and sneezing

Score	Sneezing (time/30 min)	Nasal rubbing (time/30 min)
1	1–3	1–5
2	4–10	6–15
3	> 11	> 15

### Hematoxylin–eosin (HE) staining

The formaldehyde-fixed epithelial tissues were dehydrated using gradient ethanol (70%, 80%, 90%, 95%, 100%, 5 min each), cleared twice with xylene (10 min each), embedded with paraffin, and subsequently cut into 4-μm serial sections. The sections were baked at 60 °C for 1 h and dewaxed with xylene. Following the removal of xylene using gradient ethanol, the sections were washed, stained with hematoxylin for 10 min, and washed with distilled water for a 1 min period. After being differentiated with 1% hydrochloric ethanol for 20 s, the sections were washed with distilled water for 1 min and turned blue using 1% ammonia for 30 s. The sections were then additionally stained with eosin for 3 min, dehydrated with gradient ethanol (2 min for each), cleared twice with xylene (5 min each), and mounted using neutral balsam. The samples were then observed under an optical microscope (× 40) (Olympus, Japan).

### Alcian blue (AB)-periodic acid Schiff (PAS) staining

The dewaxed epithelial tissue sections were oxidized using 1% sodium periodate solution for 10 min, stained with Schiff solution for 20 min, and differentiated with distilled water for 10 min. After nuclear staining with hematoxylin for 10 s, the sections were differentiated using 1% hydrochloric ethanol for 20 s, cleared with xylene, and mounted with neutral balsam. The sections were then observed under an optical microscope (× 40, Olympus Optical Co., Ltd., Tokyo, Japan).

### Enzyme-linked immunosorbent assay (ELISA)

ELISA was employed to detect the levels of transforming growth factor β (TGF-β), interleukin-8 (IL-8), and matrix metalloproteinase-9 (MMP-9) within the serum of the mice in each group. The antigens were diluted using a carbonate-coated loading buffer (pH = 9.6). A total of 50-μL enzyme-labeled reagent (50 μL) was added to all wells (except the blank well) and incubated at 37 °C for 1 h. Next, each reaction well was added with tetramethylbenzidine (TMB, 100 μL) substrate solution (EL0001, Huzhou InnoReagents Co., Ltd., Zhejiang, China) under conditions void of light. The reaction was then terminated through the addition of 50 μL stop buffer. Optical density (OD) was measured at 450 nm within a 15-min period using a microplate reader (SFA-680T, Nanjing Huadong Electronic Technology Company, Nanjing, China).

### Cell culture, grouping, and transfection

The epithelial tissues from control mice were treated with 0.25% trypsin (10 × volume) in a water bath at 37 °C for 10 min. The cells were then mixed with a pipette, centrifuged at 1000 r/min for 5 min with the supernatant withdrawn. DMFM/F-12 culture medium containing 10% fetal bovine serum (FBS) was added to the nasal mucosal epithelial cell pellet and mixed well to suspension. The suspension was inoculated in a culture bottle coated with polylysine at 37 °C with 5% CO_2_. The medium was replaced at regular 2-day intervals. The confluent cells were sub-cultured at a ratio of 1:2 and transfected based on their respective grouping (except for the cells in the control group).

### Dual-luciferase reporter assay

Online bioinformation analysis software microRNA.org was used to analyze target genes of miR-761 and to verify whether LCN2 was a target gene of miR-761. Dual-luciferase reporter assay was applied to further determine whether LCN2 was a target gene of miR-761. Mutant (MUT) and its complementary sites in the wildtype (WT) seed sequences were designed and inserted into pMIR-reporter vector via T4 DNA ligase following restriction enzyme digestion. Luciferase reporter plasmids with known sequences were co-transfected with miR-761 into HEK-293T cells (CRL-1415, Shanghai Xin Yu Biotech Co., Ltd., China). After 48 h of transfection, the cells were collected, cleaved, and centrifuged for 3–5 min for supernatant collection. Firefly Luciferase Assay Kit (RG005, Beyotime Biotechnology, Shanghai, China) was used to determine luciferase activity.

### RNA isolation and quantitation

Ultrapure RNA extraction kit (QIAgen GmbH, Hilden, Germany) was used to extract total RNA from nasal mucosal epithelial cells. RNA concentration was detected using a spectrophotometer (PHLES, China). The primers were designed and synthesized by Aoke Biological Technology Co. Ltd. (Beijing, China). The primer sequences are shown in Table 2. Reverse transcription was performed in accordance with the instructions of the TaqMan MicroRNA Assays Reverse Transcription Primer kit (#4366596, Thermo Scientific, Waltham, MA, USA). Fluorescence quantitative PCR was conducted as per the instructions of the SYBR® Premix Ex Taq™ II kit (RR820A, Xingzhi Biological Technology Co. Ltd., Guangzhou, China) using ABI PRISM® 7300 system (Shanghai Kun Ke instrument and Equipment Co., Ltd., Shanghai, China). U6 and glyceraldehyde-3-phosphate dehydrogenase (GAPDH) were regarded as the internal references for miR-761 and other genes, respectively. The gene expression ratio between experimental and control groups was calculated based on the 2-ΔΔCt method, ΔΔCT = ΔCt the experimental group − ΔCt the control group and ΔCt = Ct target gene − Ct internal reference. Ct was considered to reflect the amplification cycles when real-time fluorescence intensity reached the set threshold.

**Table 2:** Primer sequences for RT-qPCR

Gene	Primer sequences
miR-761	F: 5′-TTCATTCTACCCAGATGCGTT-3′
	R: 5′-GAGGATTCACGTGGAGAAGTT-3′
U6	F: 5′-ATGGGTCGAAGTCGTAGCC-3′
	R: 5′-TTCTCGGCGTCTTCTTTCTCG-3′
Twist	F: 5′-TACAGCAAGAAATCGAGCGAAG-3′
	R: 5′-GCTGAGCTTGTCAGAGGGG-3′
LCN2	F: 5′-ATGTATGGCCGGTACACTCAG-3′
	R: 5′-AACAAATGCGACATCTGGCAC-3′
E-cadherin	F: 5′-CGTGATGAAGGTCTCAGCC-3′
	R: 5′-ATGGGGGCTTCATTCAC-3′
N-cadherin	F: 5′-TAGACGAGAGGCCTATCCATGC-3′
	R: 5′-CAGCAGCTTTAAGGCCCTCAT-3′
Vimentin	F: 5′-AAAGGATCCATGTCTACCAGGTCTGTGTC-3′
	R: 5′-ACTTCTCAGCATCACGATGACTCTAGATTT-3′
GAPDH	F: 5′-GAAGGTGAAGGTCGGAGTC-3′
	R: 5′-GAAGATGGTGATGGGATTTC-3′

*RT-qPCR* reverse transcription quantitative polymerase chain reaction, *miR-761* microRNA-761, *LCN2* lipocalin 2, *GAPDH* glyceraldehyde-3-phosphate dehydrogenase, *F* forward, *R* reverse

### Western blot analysis

The nasal mucosal epithelial cells were trypsinized and centrifuged. The protein concentration was determined using bicinchoninic acid protein quantification kit (20201ES76, Yeasen Biotechnological, Shanghai, China). The protein samples were separated by sulfate-polyacrylamide gel electrophoresis, followed by transfer onto a polyvinylidene fluoride membrane. The membrane was blocked using 5% skimmed milk for 1 h. The membrane was incubated overnight with diluted primary rabbit polyclonal antibodies (Abcam Inc., Cambridge, UK) to LCN2 (ab63929, 1:1000), Twist1 (ab5887, 1:1000), E-cadherin (ab15148, 1:1000), N-cadherin (ab18203, 1:1000), and vimentin (ab137321, 1:1000). The membrane was then incubated for 1 h with secondary antibody horseradish peroxidase (HRP)-labeled goat anti-rabbit immunoglobulin G (IgG) (1:1000, Wuhan Boster Biological Technology, Ltd., Wuhan, China). The membrane was subsequently immersed in enhanced chemiluminescence (ECL) solution (Pierce, Waltham, MA, USA). The membrane was then exposed and developed accordingly. GAPDH (abs830032, Absin Bioscience Inc., Shanghai, China) was employed as the internal reference.

### 3-(4,5-Dimethylthiazol-2-yl)-2,5-diphenyltetrazolium bromide (MTT) assay

When the nasal mucosal epithelial cell density had reached approximately 80%, the cells were digested into a single-cell suspension with 0.25% pancreatin. After cell counting, the cells were seeded into a 96-well plate (3–6 × 103 cells/well, 0.2 mL/well), with six replicate wells set. After culturing for 24 h, 48 h, and 72 h, 2-μL medium containing 10% MTT solution (5 g/L) (GD-Y1317, Guduo biotechnology company, Shanghai, China) was added and incubated for 4 h. After supernatant removal, 100 mL dimethyl sulfoxide (D5879-100ML, Sigma, USA) was added and mixed to fully dissolve formazan crystals. An optical density of 490 nm was measured using a microplate reader (Nanjing DeTie laboratory equipment Co., Ltd., Nanjing, China) after which a cell viability curve was plotted.

### Flow cytometry

Annexin V-FITC/propidium iodide (PI) double staining was applied in order to detect nasal mucosal epithelial cell apoptosis. The flow cytometry detection kits were purchased from Thermo Fisher Scientific Co. Ltd. (Shanghai, China). The epithelial cells were detached with 0.25% trypsin solution, and cell concentration was adjusted to 1 × 10^6^ cells/mL. A total of 1 mL of cells were then removed for centrifugation at 1500 r/min for 10 min. After removal of the supernatant, the cells were collected and cultured at 37 °C with 5% CO_2_ for 48 h. The cells were then centrifuged and resuspended in 200 μL binding buffer. Annexin V-FITC (10 μL) and 5 μL propidium iodide (PI) were added and incubated under dark conditions for 15 min, followed by the addition of 300 μL binding buffer. FACSCalibur flow cytometer (BD Bioscience, USA) was used to determine cell apoptosis at 488 nm.

### Statistical analysis

All statistical analyses were conducted using SPSS 21.0 (IBM Corp. Armonk, NY, USA). Measurement data were expressed as mean ± standard deviation. Data conforming to normal distribution and homogeneous variance as well as the data between two groups were compared by *t* test while the data among multiple groups were compared by one-way analysis of variance (ANOVA) followed by Tukey’s post hoc test. Data at different time points were analyzed using repeated measures ANOVA followed by Bonferroni’s post hoc test. Statistical differences were considered to be significant when *p* < 0.05.

## Results

### miR-761 negatively regulates the expression of the target gene LCN2

**Fig. 1:**
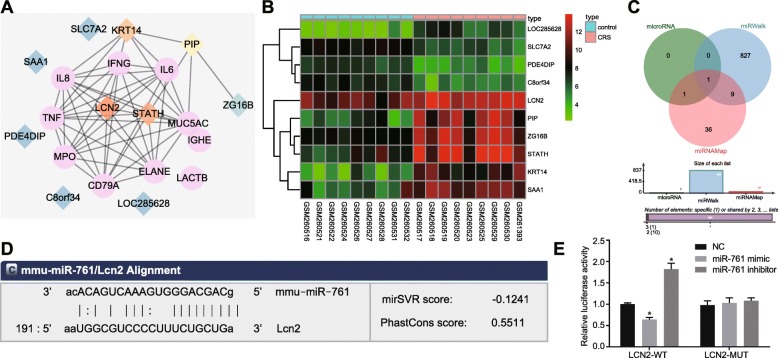
Bioinformatics analysis of the molecular mechanisms of CRS. **a** Interaction network of CRS genes and DEGs obtained from GSE10406 chip. Pink circle denotes CRS genes while diamond denotes DEGs. Orange represents a large amount of interacting gens while blue represents a smaller amount. **b** Heat map of the first 10 DEGs from GSE10406 chip. The abscissa was sample number and ordinate was DEGs. Each rectangle represented an expression of a sample. Red indicates high expression while green indicates low expression. **c** Venn diagram showing miRs that may regulate LCN2 from microRNA, miRWalk, and miRNAMap databases. **d** Targeting relation between miR-761 and LCN2 predicted using TargetScan database (http://www.targetscan.org/). **e** Targeting relation between miR-761 and LCN2 validated using dual-luciferase reporter gene assay. Data is represented as mean ± standard deviation. *p < 0.05 compared with the NC group. Data were analyzed by independent sample *t* test. The cellular experiment was repeated three times

Based on the microarray analysis, a total of 63 DEGs were identified in CRS with |LogFoldChange| > 2 and *adj. P. Val* as threshold. Next, the CRS-related genes were identified through the Digsee dataset, in which the top 10 genes (IL8, TNF, IGHE, ELANE, LACTB, IL6, IFNG, CD79A, MPO, and MUC5AC) were screened as the CRS genes. The 20 genes were included in the String database for gene interaction analysis. Figure 1a illustrates the interaction network constructed for these 20 genes. Except for LACTB and IGHE, the remaining CRS genes were found to be closely correlated. LCN2 was found to interact with multiple CRS genes in the interaction network. The heat map of the first 10 DEGs from the GSE10406 chip is shown in Fig. 1b. LCN2 was highly expressed in CRS patients than controls, highlighting the likely involvement of LCN2 in CRS. LCN2/Twist1 signaling pathway was associated with liver cancer [16]. We subsequently sought to investigate the effects of the LCN2/Twist1 signaling pathway in CRS. Venn diagram revealed that microRNA predicted 2, miRWalk 837, and miRNAMap 47 miRs could potentially work to regulate LCN2 (Fig. 1c). The mmu-miR-761 was predicted by all three databases, suggesting miR-761 might target LCN2. Hence, we revealed that miR-761 could play a role in CRS via the LCN2/Twist1 signaling pathway by targeting LCN2.

Next, in order to evaluate the targeting relationship between miR-761 and LCN2, the TargetScan database was used to analyze the specific binding sites between LCN2 3′UTR and miR-761 sequences, the results of which revealed that LCN2 was indeed the target of miR-761 (Fig. 1d). Next, the targeting relation was further verified based on the results of the dual-luciferase reporter gene assay (Fig. 1e), which revealed that the transfection of miR-761 mimic effectively inhibited the luminescent signal of LCN2-WT-3′UTR, while transfection of the miR-761 inhibitor notably strengthened the luminescent signal of LCN2-WT-3′UTR. However, the exact treatment triggered no significant change in LCN2-MUT-3′UTR. The aforementioned results indicated that miR-761 was able to specifically bind to LCN2.

### miR-761 overexpression or LCN2 silencing improves the CRS symptoms

**Fig. 2:**
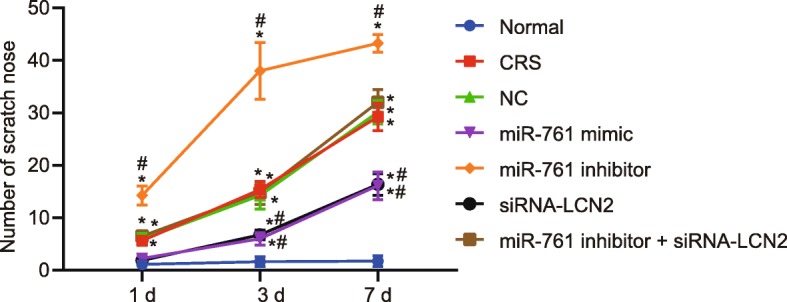
Statistics for nasal rubbing of mice. Each group of data is represented by mean ± standard deviation. *p < 0.05 compared with the normal group. ^#^*p* < 0.05 compared with the NC group. Data at different time were compared using repeated measures ANOVA followed by Bonferroni’s post hoc test; *n* = 8

**Fig. 3:**
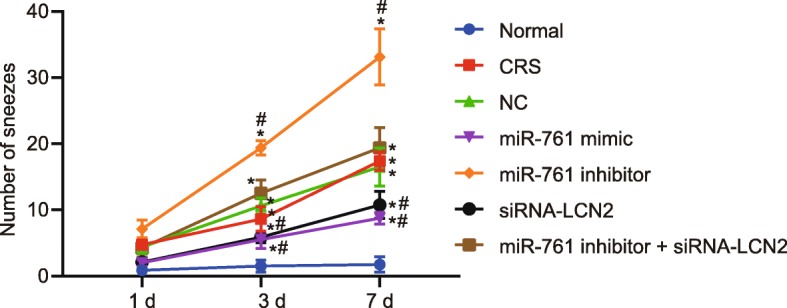
Statistics for sneezing of mice. Each group of data is represented by mean ± standard deviation. *p < 0.05 compared with the normal group. ^#^*p* < 0.05 compared with the NC group. Data at different time were compared using repeated measures ANOVA followed by Bonferroni’s post hoc test; *n* = 8

Before CRS modeling, all the mice were confirmed to be in good physical and mental condition with glossy hair and regular diets. One week following CRS modeling, the mice in the normal group exhibited the same condition recorded prior to modeling. In contrast, the mice in the CRS, and NC groups as well as the co-transfection of miR-761 inhibitor and si-LCN2 exhibited an elevated frequency of sneezing and nasal rubbing, accompanied by a greater severity of rhinorrhea and secretion. However, a diminished frequency of sneezing and nasal rubbing, alleviated rhinorrhea, and rare secretions were identified among the mice transfected with miR-761 mimic and the mice transfected with si-LCN2. The transfection of miR-761 inhibitor drastically stimulated the frequency of sneezing and nasal rubbing, leading to more severe symptoms of rhinorrhea and secretions (Figs. 2 and 3).

### Depleted miR-761 expression stimulates inflammation and aggravates CRS symptoms

**Fig. 4:**
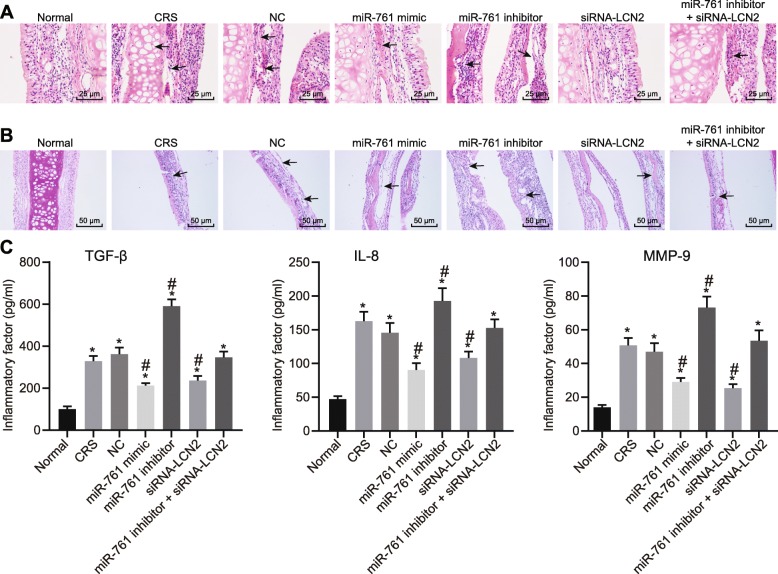
The nasal mucosal tissues of mice are analyzed through histological study. **a** HE staining (× 100) on nasal mucosal tissues of mice. **b** AB-PAS staining on nasal mucosal epithelial gland of mice (× 400). **c** Inflammatory factors in the serum of mice detected using ELISA. Data is represented as mean ± standard deviation. *p < 0.05 compared with the normal group. ^#^*p* < 0.05 compared with the NC group. One-way ANOVA was applied for comparison among multiple groups followed by Tukey’s post hoc test; *n* = 8

HE staining was performed in order to further explore the histopathology of the mouse nasal mucosal tissues. The results (Fig. 4a) obtained revealed pseudostratified ciliated epithelia that were uniformly arranged, structurally healthy with no notable inflammatory cell infiltration in the submucosa as well as scarcely diffused distributed goblet cells in the control mice. However, in CRS mice, the paranasal sinus mucosa was observed to have marked chronic inflammation with lymphocyte and plasma component infiltration, disorganized mucosal epithelial arrangement with tissue peeled off and necrosis, as well as fibrosis within the intraepithelial goblet cells and submucosal glands. In comparison to the CRS group, paranasal sinus mucosa from mice in NC and miR-761 inhibitor + siRNA-LCN2 groups displayed no significant difference in regard to tissue morphology. Mice in the miR-761 mimic and siRNA-LCN2 groups exhibited alleviated levels of inflammation, while the miR-761 inhibitor group was found to have increased inflammation when compared to the CRS mice. AB-PAS staining revealed that both the epithelia and glands in the paranasal sinus mucosa were enlarged with increased goblet cells in CRS and NC when compared to control mice (Fig. 4b). Mice in the miR-761 mimic and siRNA-LCN2 groups exhibited less severe morphological changes when compared to the changes in the CRS group, while the miR-761 inhibitor group exhibited more distinct changes. The aforementioned findings provided evidence that the CRS mouse model had indeed been successfully established, while suggesting that activation of miR-761 or silencing LCN2 may be beneficial to the pathological changes of CRS.

ELISA was conducted to evaluate the inflammatory factors in the serum of mice (Fig. 4c). The expression of TGF-β, IL-8, and MMP-9 was elevated in all groups except in the normal group. When compared to the NC group, TGF-β, IL-8, and MMP-9 levels were decreased in the miR-761 mimic and si-LCN2 groups. However, the expression of TGF-β, IL-8, and MMP-9 in the miR-761 group was higher than that in the NC group.

### Upregulated LCN2 activates LCN2/Twist1 signaling pathway in CRS

**Fig. 5:**
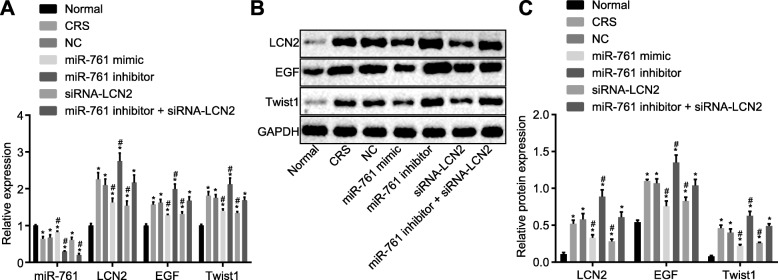
miR-761 targets LCN2 and regulates the LCN2-mediated LCN2/Twist1 signaling pathway. **a** miR-761, LCN2, EGF and Twist1 mRNA expression in nasal mucosal cells detected using RT-qPCR. **b** miR-761, LCN2, EGF and Twist1 protein expression in nasal mucosal cells detected using western blot analysis. **c** statistical results of the western blot shown in Panel **b**. Data is represented as mean ± standard deviation. *p < 0.05 compared with the normal group. ^#^*p* < 0.05 compared with the NC group. One-way ANOVA was applied for comparison among multiple groups followed by Tukey’s post hoc test. The cellular experiment was repeated three times

Next, in order to ascertain as to whether the miR-761/LCN2 mechanism affected the LCN2/Twist1 signaling pathway, the expression of miR-761, LCN2, EGF, and Twist1 was evaluated via RT-qPCR and western blot analysis methods (Fig. 5a–c). Next, the protein expression of miR-761 was found to be significantly diminished while the expression of LCN2 and Twist1 exhibited elevated levels in all groups when compared to the normal group (*p* < 0.05). There was no marked difference detected regarding the expression of miR-761 between the CRS and NC groups (*p* > 0.05). In comparison to the NC group, the miR-761 mimic and si-LCN2 groups showed decreased LCN2 and Twist1 expressions (*p* < 0.05). In the miR-761 inhibitor group, the expression of LCN2 and Twist1 was elevated while that of miR-761 was decreased when compared to CRS. miR-761 expression was reduced in the miR-761 inhibitor + si-LCN2 group (*p* < 0.05) while LCN2 and Twist1 were unchanged (*p* > 0.05). Based on the results, we concluded that overexpressed miR-761 and silenced LCN2 inactivated the LCN2/Twist1 signaling pathway.

### Overexpression of miR-761 and depletion of LCN2 suppresses EMT in CRS

**Fig. 6:**
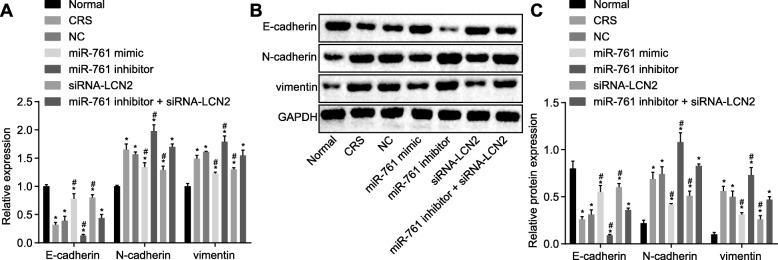
Effects of miR-761 overexpression or LCN2 silencing on expressions of mediators that suppresses EMT in CRS. **a** mRNA expression of E-cadherin, N-cadherin, and vimentin determined by RT-qPCR. **b** Protein expression of E-cadherin, N-cadherin, and vimentin examined by western blot analysis. **c** Statistical results of the western blot shown in panel **b**. Data is represented as mean ± standard deviation. *p < 0.05 compared with the normal group. ^#^*p* < 0.05 compared with the NC group. One-way ANOVA was applied for comparison among multiple groups followed by Tukey’s post hoc test. The cellular experiment was repeated three times

In order to ascertain as to whether aberrantly expressed miR-761 or LCN2 regulated EMT, the expression of E-cadherin, N-cadherin, and vimentin was assessed using RT-qPCR and western blot analysis (Fig. 6a, b). The results revealed that the mRNA and protein expressions of E-cadherin were remarkably decreased while those of N-cadherin and vimentin were elevated in nasal mucosal epithelial cells in all groups compared to the normal group (*p* < 0.05). There was no significant difference among all three peptides between CRS and NC groups (*p* > 0.05). Compared with the NC group, the miR-761 mimic and siRNA-LCN2 groups had decreased expression of N-cadherin and vimentin, but increased E-cadherin. In the miR-761 inhibitor group, N-cadherin and vimentin expressions were increased while E-cadherin was decreased when compared to CRS. All three peptides did not reflect any differences between miR-761 inhibitor + siRNA- LCN2 and CRS group (all *p* < 0.05). These findings provided evidence that miR-761 overexpression LCN2 silencing may contribute to inhibited EMT in CRS.

### miR-761 overexpression or LCN2 silencing promotes cell viability and inhibits cell apoptosis

**Fig. 7:**
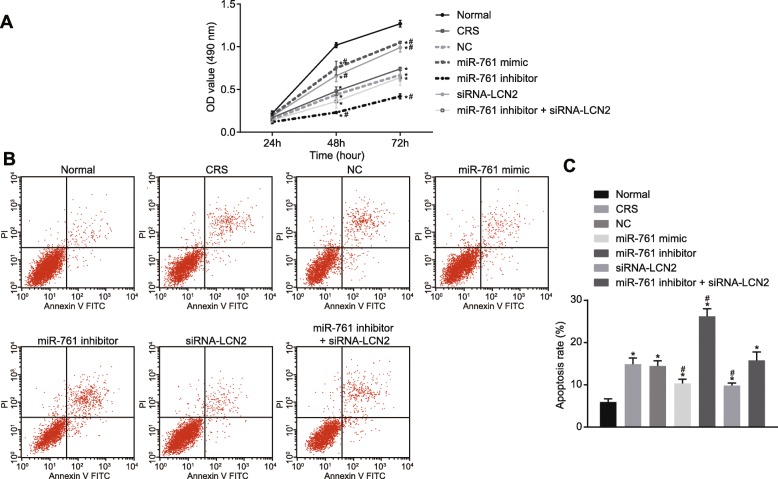
Effects of miR-761 overexpression or LCN2 silencing on cell viability and cell apoptosis. **a** Cell viability at different time points in nasal mucosal cells determined using MTT assay. **b** Cell apoptosis in each group examined by flow cytometry. **c** Statistical results of the cell apoptosis in each group in panel **b**. *p < 0.05 compared with the normal group. ^#^*p* < 0.05 compared with the NC group. One-way ANOVA was applied for comparison among multiple groups followed by Tukey’s post hoc test. The cellular experiment was repeated three times

In order to elucidate the effects of altered expression of miR-761 or LCN2 on cell viability and apoptosis, MTT assay was performed to measure cell viability at 24 h, 48 h, and 72 h (Fig. 7a). Cells in all groups were similar after 24 h (*p* > 0.05). However, at 48 h and 72 h, decreased cell viability was observed in all groups when compared to the normal group (*p* < 0.05). There was no difference in cell proliferation between the CRS and NC groups (*p >* 0.05). In comparison with the CRS and NC groups, cell viability in the miR-761 mimic and siRNA-LCN2 groups was elevated (*p* < 0.05) but decreased in the miR-761 inhibitor group (*p* < 0.05). Significant differences were detected between, CRS, NC, and miR-761 inhibitor + siRNA-LCN2 groups (*p* > 0.05). These findings demonstrated that miR-761 overexpression and LCN2 silencing promoted cell viability.

Flow cytometry was performed to determine cell apoptosis (Fig. 7b, c). The apoptosis rates of the normal group; the CRS group; the NC group; the transfection of miR-761 mimic, miR-761 inhibitor, si-LCN2; and co-transfection of miR-761 inhibitor and si-LCN2 were 5.92 ± 0.75%, 14.85 ± 1.52%, 14.47 ± 1.19%, 10.32 ± 0.98%, 26.18 ± 1.84%, 9.83 ± 0.61%, and 15.78 ± 2.01%, respectively. The rate of apoptosis was significantly increased in all groups when compared to the normal group. No significant difference regarding apoptosis was found between the CRS and NC groups (*p* > 0.05). Compared with the CRS and NC groups, apoptosis was reduced in the miR-761 mimic and siRNA-LCN2 groups (*p* < 0.05) but increased in the miR-761 inhibitor group (*p* < 0.05). No difference was identified in the miR-761 inhibitor + siRNA-LCN2 group when compared to CRS. The above findings confirmed that upregulated miR-761 and silenced LCN2 gene expression could inhibit cell apoptosis.

## Discussion

CRS is a condition triggered by various extrinsic factors including microbial infections or intrinsic factors that predispose patients to infection or inappropriate and excessive inflammatory responses [19]. Partial EMT has been identified to occur in certain patients suffering from CRS, which may trigger the pathogenesis of nasal polyps [20]. EMT has been considered to be a novel therapeutic target in tissue remodeling in the setting of various chronic airway diseases [21]. Existing literature has highlighted the involvement of miRNA dysregulation in several inflammatory diseases, including CRS [22]. Hence, the investigation into miRNAs associated with inflammation and EMT from a CRS perspective is a crucial step in identifying new therapeutic targets for CRS. During the current study, we identified that overexpressed miR-761 inhibited LCN2 gene as well as the LCN2/Twist1 signaling pathway, ultimately alleviating chronic inflammation and remodeling of nasal mucosa in CRS mice.

A notable finding of the present study revealed that the overexpression of miR-761 or silencing of LCN2 alleviated inflammation, which is a widely documented hallmark feature of CRS. This finding was consistent with previous reports that highlighted the link between miRs and inflammation in CRS [23]. Additionally, miR-124, for instance, has been demonstrated to target aryl hydrocarbon receptor to regulate inflammatory response in CRS [24]. Moreover, reports have suggested that LCN2 is highly expressed in episodes of inflammation and anti-bacterial responses [25] while silencing of LCN2 has been shown to alleviate inflammation in adipose tissues [26]. The aforementioned findings were in line with the observations of our study whereby miR-761 and LCN2 were found to play a role in the inflammatory response associated with CRS.

Cell proliferation and apoptosis are fundamental events in tissue remodeling. Our results revealed that miR-761 promoted cell proliferation and suppressed apoptosis by negatively regulated LCN2. Consistent with a previous study, it was shown that miR-761 suppressed cardiomyocyte apoptosis [27]. On the other hand, elevated LCN2 has been shown to promote cell differentiation and invasion in esophageal squamous cell carcinoma [28] and depletion of LCN2 suppressed renal tubules apoptosis [29]. It is worth mentioning that, although the aforementioned studies were performed in other tissues, their findings were in line with our results, whereby miR-761-mediated inhibition of LCN2 is likely responsible for cell proliferation and reduced apoptosis in CRS mice.

Furthermore, our results provided evidence that the overexpression of miR-761 suppressed the remodeling of nasal mucosa and EMT in CRS mice by inactivating the LCN2/Twist1 signaling pathway. E-cadherin, N-cadherin, and Twist all represent distinct markers of the EMT process. In epithelial cells from nasal polyps in CRS, an opposite trend of E-cadherin and N-cadherin expression was identified [20], which was consistent with our finding that miR-761 downregulated N-cadherin but upregulated E-cadherin. In addition, a recent report showcased that the downregulation of E-cadherin and upregulation of vimentin were related to the elevation of neutrophil gelatinase-associated lipocalin [30], which was in line with our results that the depletion of LCN2 led to an increase in E-cadherin and reduced vimentin. LCN2 has been previously shown to reversely regulate EMT via the LCN2/Twist signaling pathway in hepatocellular carcinoma [18]. Taken together, the results of our study revealed that the overexpression of miR-761 or silencing of LCN2 led to a reduction in the expression of Twist1, highlighting the likelihood that miR-761 may reduce EMT by suppressing LCN2 and inactivating the LCN2/Twist signaling pathway.

## Conclusions

**Fig. 8:**
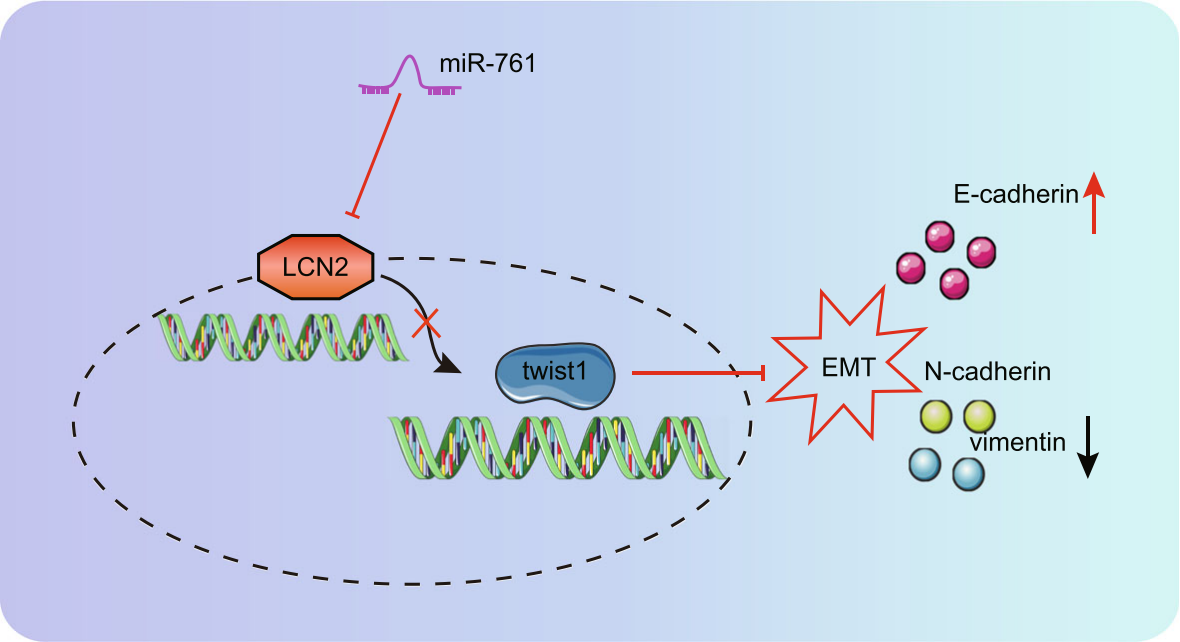
Schematic diagram showing potential involvement of miR-761 inhibiting EMT in CRS mice through targeting LCN2 in the LCN2/Twist1 signaling pathway

In conclusion, the key findings of the current study present evidence demonstrating that miR-761 suppresses the remodeling of nasal mucosa and EMT in CRS mice models by downregulating LCN2 and inactivating the LCN2/Twist1 signaling pathway (Fig. 8). Further studies are warranted in order to further evaluate the potential of miR-761 and to ascertain its true therapeutic value for CRS.

## Data Availability

The datasets generated/analyzed during the current study are available.

## Abbreviations

CRS: Chronic rhinosinusitis
LCN2: Lipocalin 2
DEGs: Differentially expressed genes
HE: Hematoxylin–eosin
AB: Alcian blue
PAS: Periodic acid Schiff
